# MALDI-TOF Mass Spectrometry Technology as a Tool for the Rapid Diagnosis of Antimicrobial Resistance in Bacteria

**DOI:** 10.3390/antibiotics10080982

**Published:** 2021-08-14

**Authors:** Eun-Jeong Yoon, Seok Hoon Jeong

**Affiliations:** 1 Division of Antimicrobial Resistance, Center for Infectious Diseases, National Institute of Health, Korea Disease Control and Prevention Agency, Cheongju-si 28159, Korea; ejyoon3@korea.kr; 2 Department of Laboratory Medicine, Yonsei University College of Medicine, Seoul 06273, Korea; 3 Research Institute of Bacterial Resistance, Yonsei University College of Medicine, Seoul 06273, Korea

**Keywords:** MALDI-TOF MS, antimicrobial resistance, rapid diagnostics

## Abstract

Species identification by using matrix-assisted laser desorption/ionization time-of-flight mass spectrometry (MALDI-TOF MS) is a routine diagnostic process for infectious diseases in current clinical settings. The rapid, low-cost, and simple to conduct methodology is expanding its application in clinical microbiology laboratories to diagnose the antimicrobial resistance (AMR) in microorganisms. Primarily, antimicrobial susceptibility testing is able to be carried out either by comparing the area under curve of MALDI spectra of bacteria grown in media with antimicrobial drugs or by identifying the shift peaks of bacteria grown in media including ^13^C isotope with antimicrobial drugs. Secondly, the antimicrobial resistance is able to be determined through identifying (i) the antimicrobial-resistant clonal groups based on the fingerprints of the clone, (ii) the shift peak of the modified antimicrobial drug, which is inactivated by the resistance determinant, (iii) the shift peak of the modified antimicrobial target, (iv) the peak specific for the antimicrobial determinant, and (v) the biomarkers that are coproduced proteins with AMR determinants. This review aims to present the current usage of the MALDI-TOF MS technique for diagnosing antimicrobial resistance in bacteria, varied approaches for AMR diagnostics using the methodology, and the future applications of the methods for the accurate and rapid identification of AMR in infection-causing bacterial pathogens.

## 1. Introduction

Antimicrobial drugs have extended the human lifespan by saving lives from acquiring infectious diseases [1]. However, drug usage has resulted in a drastic increase in antimicrobial resistance (AMR), including the emergence of resistance to multiple antimicrobial drugs; therefore, the rapid and accurate diagnosis of antimicrobial susceptibility is encouraged to optimize the therapeutic strategy for patients with infectious diseases and control infection in healthcare settings [2]. In terms of the AMR of infection-causing bacteria, physicians need at least three pieces of information from clinical microbiology laboratories: (i) whether the patient is infected by bacteria, (ii) if so, with which bacterial species, and (iii) which antimicrobial regimen will treat the infection [3]. Rapid and accurate diagnostics help physicians choose an appropriate drug regimen promptly, improve patient outcomes, decrease patient hospitalization duration, and reduce the potential for pathogen spread [4].

The gold standards of antimicrobial susceptibility testing (AST) are based on bacterial growth methods that determine the inhibition zone diameter around antimicrobial disks on an agar surface or the minimum inhibitory concentration of an antimicrobial drug [5] following the guidelines standardized by the Clinical Laboratory Standards Institutes (USA), the British Society for Antimicrobial Chemotherapy (UK), la Société Française de Microbiologie (France), the European committee on Antimicrobial Susceptibility Testing (EU), etc. The primary defect of these methods is that it takes days to produce results, which impedes the rapid elaboration of proper treatment strategies for the patient. The identification of AMR determinants, mostly based on genotype-determining molecular biological methodologies, also has several limitations. First, the genotype does not always correspond to the phenotype [6]. Some AMR genes need to be induced for expression, and certain, but not rare, genetic alterations block the proper production of the particular protein(s) that confer resistance to the antimicrobial drug. Second, detection of new, emerging AMR determinants is impractical by genotyping. Third, coupling the AMR genotype with the AMR phenotype of a bacterial strain requires a deep understanding of the field [7], which means that this methodology needs experienced experts for data interpretation. Finally, the technique is laborious and cost-inefficient, which impedes its wide use in clinical microbiology laboratories.

Among the various techniques that have been suggested for rapid AMR diagnostics, the matrix-assisted laser desorption ionization time-of-flight mass spectrometry (MALDI-TOF MS) method, which is already widely used in clinical settings for the rapid species identification of organisms with medical importance, has been considered a supplement for the growth-based AST [8]. This review aims to show varied approaches of MALDI-TOF MS to rapidly determine AMR and develop an optimal treatment strategy for patients with infectious diseases.

## 2. Application of MALDI-TOF MS in Clinical Microbiology Laboratories

The identification of bacterial species based on peptide spectra obtained from mass spectrometry (MS) was first proposed in the 1970s [9]. However, directly exposing the fragile bacterial samples to a powerful laser for desorption and ionization of the sample was a challenging step that needed improvement. In the mid-1980s, Koichi Tanaka developed a soft desorption ionization method using ultrafine metal powder and glycerol that enabled MS analysis of biological macromolecules [10], and he was awarded the Nobel Prize in Chemistry in 2002 for this work. Quite coincidently, Franz Hillenkamp and Michael Karas reported another soft desorption ionization methodology using an organic compound matrix [11] and finally, the matrix-assisted laser desorption ionization (MALDI) methodology was conceived. MALDI-TOF MS was successfully introduced in clinical laboratories in the early 2000s as a tool to identify particular species of organisms based on speed (less than a minute per sample) and accurate identification of clinically relevant pathogens, especially bacteria and fungi, with cost-effective benefits. Currently, the method of applying bacterial and fungal colonies directly onto a MALDI target plate with matrices for cocrystallization has become the standard protocol for the identification of bacterial and fungal species in clinical laboratories [12].

**Figure 1:**
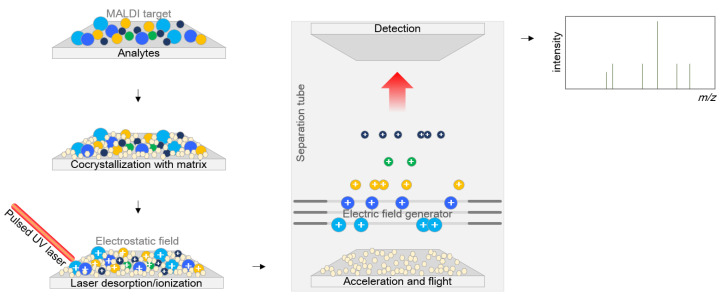
Mechanism of matrix-assisted laser desorption/ionization time-of-flight mass spectrometry. Analytes are first cocrystallized within the matrix, and energy is beamed down through a laser beam to ionize the analyte to generate separate protonated ions. These ions are accelerated at a fixed potential and separated from each other on the basis of their weights when they fly along the separation tube. The flight time of each ion is measured at the detection panel placed at the end of the flight tube.

The matrices consist of crystallizable molecules as a mixed solution in water or organic solvents. Various matrix compounds are now available, and additional compounds are under development [13] with three indispensable requisites: (i) having an appropriate molecular weight, which is low enough to ensure easy vaporization and large enough not to be entirely evaporated in the vacuum separation tube while standing in the apparatus, (ii) having a strong optical absorption in either the ultraviolet or infrared region to absorb laser irradiation rapidly and efficiently, and (iii) being an excellent proton donor that drives the ionization of the analyte. Basically, the matrix plays two roles, both as a helper for crystallization for efficient ionization and as a buffer between the sample and the powerful laser. The sample entrapped within the matrix is cocrystallized with the matrix, and the energy is applied to the sample as a laser beam to ionize the analyte, generating separate protonated ions. These ions are accelerated at a fixed potential and separated from each other on the basis of their weight during flight along the flight tube. The time of flight is measured by determining the time to reach the detection panel at the end of the flight tube (Figure 1).

MALDI-TOF MS has become a welcome addition to routine microbiology laboratory practice. The most widely used analytical strategy using this technique is analysis of the fingerprint spectra in the range of 2000 to 20000 *m*/*z*, reflecting the composite proteome of a bacterial cell [14]. The identification of an infection-causing microorganism is possible through this methodology. Before the introduction of the MALDI-TOF MS-based identification method in clinical laboratories, biochemical tests were used for the identification of bacteria and fungi. The process is very complex and requires multiple steps. Moreover, the method needs an individualized approach by organism type, and the results are often ambiguous. Clinical microbiologists need to be trained to conscientiously perform and interpret the colony phenotypes and Gram-stain morphology of bacteria growing on solid media to determine the appropriate biochemical tests and additional core gene DNA sequencing. However, through the application of MALDI-TOF MS, most of the colonies are directly applied for identification devoid of any preparation steps, and accurate identification is available in minutes without experiential knowledge. In addition, with a few exceptions of hazardous organisms requiring careful handling, complex decision-making processes are not required. Testing can be performed from a single colony on primary culture plates. Complicated DNA sequencing and its expenses are avoided for organism identification, the amount of waste decreases, and training of laboratory technicians and excess labor are eliminated.

A decade of MALDI-TOF MS experience with bacterial species identification in a clinical microbiology laboratory has indicated the following three efficiencies of this method compared to traditional biochemical methods [15]. MALDI-TOF MS and traditional biochemical methods are able to identify 36 and 19 bacterial species per 10000 isolates, respectively. Supplemental genotypic identification using 16S rRNA sequences is required for 4.5 and 35.2 isolates per 10000 isolates with MALDI-TOF MS and biochemical methods, respectively. Finally, MALDI-TOF MS reduced the time for identification by 55-fold and the cost by 5-fold compared with biochemical tests.

Culture-independent direct application of clinical specimens to MALDI-TOF MS analysis to identify the infection-causing pathogen has been regarded as impractical because of the various extra molecules that can inhibit the ability to obtain clear spectra derived from the pathogen. However, direct application of urine specimens to MALDI-TOF MS after simple processing has become possible, owing to the high concentration of bacteria in these specimens [16]. Polymicrobial infections [17] and abundant proteins of human origin in urine specimens, such as the antimicrobial peptides α-defensins, which are released from neutrophils to eliminate urinary tract infections [18], have been the major limitations of the direct application of urine specimens. Various processing methods to prepare urine specimens suitable for MALDI-TOF MS analysis have thus been proposed [19] and are still under development. The direct MALDI-TOF MS application of clinical specimens other than urine, such as cerebrospinal fluid [19,20] and blood [21], has also been attempted and has presented results that seem not feasible, but possible.

## 3. AST by Using the MALDI-TOF MS

The major limitations of growth-based AST, which have time and labor dependencies, have been considered to be solved by using the MALDI-TOF MS-based approach. A microdroplet assay, Direct-on-Target Microdroplet Growth Assay, was developed to overcome the weak point of the traditional AST method by using MALDI-TOF MS [22]. Basically, this method is based on comparative analysis of the spectral intensity of bacteria grown in a droplet-amount of media with or without an antimicrobial drug. Reduction in intensity after drug administration compared to that in the absence of drug is assumed to be the antimicrobial drug susceptibility [23].

An approach using isotope-labeled nutrients, usually ^13^C-labeled amino acids, has been able to give clear results by shift spectra [24,25]. Commercial media composed of 98% ^13^C or isotope-labeled lysine (^13^C_615_N_2_-L-lysine) have been used for the methods. Bacterial growth of an isolate is monitored by the MALDI spectrum after a few hours of incubation of the isolate in the labeled medium with or without an antimicrobial. Comparative analysis of the numbers and intensities of the isotope-mediated shift peaks after growth without the antimicrobial drug to those after growth with the drug may explain drug susceptibility.

These methods were developed as an alternative method for manual AST; however, a few limitations remain. These include finding a proper single concentration of each antimicrobial drug for testing for each bacterial species, optimization of both a universal inoculum size and a universal incubation time for each bacterial species, and removal of the liquid medium with the least amount of loss of the biomass.

## 4. Detecting the Antimicrobial Resistance

### 4.1. Identification of Antimicrobial-Resistant Clonal Groups

**Figure 2:**
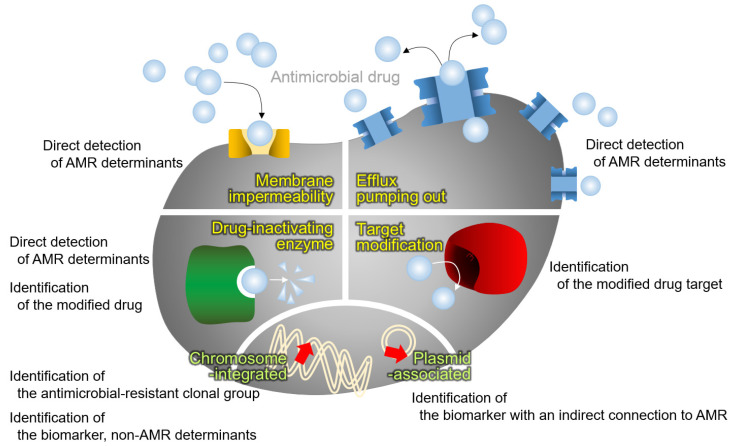
MALDI-TOF MS approaches for AMR detection. Four mechanisms of antimicrobial resistance (in yellow), i.e., membrane impermeability by loss or modification of the outer membrane porin, overproduction of the drug efflux pumping out system, antimicrobial drug modification by the drug-inactivating enzyme, and modification of the drug target, and the location of the genes for AMR (in lime), i.e., integrated in chromosome and located in plasmid, are presented with the relevant approach for AMR diagnostics using the MALDI-TOF MS.

As a coherent approach for species identification of microorganisms through the whole-cell proteome, MALDI-TOF MS using protein fingerprints of well-known antimicrobial-resistant clonal groups has been attempted (Figure 2). Basically, this strategy is achievable by means of the concept of bottom-up proteomics using a very large set of bacterial strains.

The most successful results to detect the antimicrobial-resistant clonal group were achieved with *Bacteroides fragilis* [26]. This species is an important Gram-negative anaerobic pathogen, and a cluster of *B. fragilis*, the so-called division II, carries the *cfiA* gene encoding a metallo-beta-lactamase (MBL). The intrinsic gene is overexpressed when an insertion sequence is integrated upstream of the gene, providing a strong promoter. Then, CfiA MBL confers resistance against nearly all beta-lactam drugs to the bacterial host. Through an extensive screening of *B. fragilis*, the *cfiA*-positive division II strains were distinguished from the *cfiA*-negative strains by spectral shifts between 4000–5500 *m*/*z*, and the results were satisfactory.

Distinguishing methicillin-resistant *Staphylococcus aureus* (MRSA) from methicillin-susceptible *S. aureus* (MSSA) is a matter of popular interest. One of the common strain-typing methods, multilocus sequence typing (MLST), is closely associated with Staphylococcal cassette chromosome *mec* (SCC*mec*) types, including the *mecA* gene in MRSA. Since MRSA clones of specific sequence types are closely associated with certain types of SCC*mec*, clonal grouping by using the whole-cell proteome spectral fingerprints by MALDI-TOF MS was carried out [27]. Most of the epidemic MRSA isolates that cause human infection belong to specific MLST clonal complexes (CCs), including CC5, CC8, CC22, CC30, and CC45 [27,28], and CC97 and CC398 have been identified in MRSA infections associated with livestock [29]. Thirteen characteristic peaks in the MALDI-TOF MS spectrum have been reported to be useful to distinguish MRSA from MSSA [30]. A study to distinguish between the MRSA groups of SCC*mec* types I–III, which are usually associated with hospital-associated infections, from those of SCC*mec* types IV and V, which are usually associated with community-associated infections, was carried out using a group-specific peak in the range of 1000–4000 *m*/*z*, and peaks at 1772 *m*/*z* and 1792 *m*/*z* from the phenol-soluble portion were identified as being specific for the former MRSA clones [31].

Vancomycin-resistant *Enterococcus* (VRE) was another target for identification through this strategy. The *vanA* gene-carrying *Enterococcus faecium* clinical strains, which are clustered into a group via a programmed algorithm based on the MALDI-TOF MS spectra, were detectable; however, this could not be demonstrated again in other studies [32]. Detection of the *vanB* gene-carrying VRE lineage was attempted by comparing the MALDI-TOF MS spectra with WGS data [33]. Similar to *vanA*-type VRE, this discriminatory power has never been demonstrated in another study, even though the methodology was proven through external validation with great diagnostic power, meaning that the detected VREs in the study were endemic clones in the region.

In addition, vancomycin-intermediate *S. aureus* (VISA) [34], MRSA [35], and carbapenem-resistant *Klebsiella pneumoniae* [36] were also subjected to studies to identify AMR through clonal lineage detection, often using machine learning technology [37]. However, none of these studies have been proven to be useful to the public.

### 4.2. Identification of a Modified Antimicrobial Drug

AMR in bacteria is often conferred by enzymes that inactivate antimicrobial drugs, either by hydrolyzing or by transferring acyl, phosphoryl, thiol, nucleotidyl, ADP-ribosyl, and/or glycosyl groups to the drug. Enzymes with hydrolysis activity, which need water as a cosubstrate, are beta-lactamases and macrolide esterases [38]. Additionally, the transferase enzyme group is composed of six enzymes depending on the group to be transferred, i.e., acyltransferases, phosphotransferases, thioltransferases, nucleotidyltransferases, ADP-ribosyltransferases, and glycosyltransferases [38]. As the modified antimicrobial drugs change their molecular weights through the molecules added, antimicrobial resistance is able to be identified by detecting the peak shift using the MALDI-TOF MS methodology (Figure 2).

Beta-lactamases are biochemically classified into two groups: MBL and serine beta-lactamase. The MBL, which needs Zn^2+^ ions at the active site to cleave the beta-lactam ring, uses metal-ligating amino acids together with polarized water molecules. The serine beta-lactamase, whose active site includes a serine residue, hydrolyzes the beta-lactam ring by acylation/deacylation mechanisms [39]. Both of these beta-lactamases carry out enzymatic cleavage of the beta-lactam ring with the aid of polarized water molecules, resulting in the disappearance of the original mass peak and the appearance of a new peak that is 18-Da greater in molecular mass due to the water molecule. The shift peak is targeted by MALDI-TOF MS for detection. For the analysis, a total of 30–120 min is needed for incubation of the target bacteria with the beta-lactam drug, with the exception of OXA-48-type beta-lactamases, which have a slow hydrolysis rate and require up to 240 min of incubation. Commercial software, i.e., the MBT-STAR-BL assay from Bruker, is available [40]. Of note, the shift spectra are found in a slightly different site by the selected matrix. For instance, the α-cyano-4-hydroxycinnamic acid matrix eliminated carbon dioxide and water molecules after hydrolysis, resulting in a shift of −44 *m*/*z* for the modified carbapenem rather than the +18-*m*/*z* shift from the additional water molecule [41]. Accordingly, the characteristic shift peaks at the +18 *m*/*z* and −44 *m*/*z* sites are useful to identify modified beta-lactams.

Aminoglycoside-modifying enzymes are grouped into three types: acyltransferases, phosphotransferases, and nucleotidyltransferases [42]. The three transferase reactions are complex compared to the hydrolysis activity of beta-lactamases, because additional cofactors, such as ATP, acyl-CoA, NAD+, UDP glucose, and glutathione, are required for enzymatic activity. Acyltransferases show different preferences for the CoA substrate to modify different aminoglycosides. Several attempts have been made to establish an equal mass spectrometry-based assay for the detection of resistance against aminoglycosides. However, biosynthetic or semisynthetic drugs are often a mixture of different molecules that vary in their side chains [43], and modifications by aminoglycoside-modifying enzymes vary by the moiety introduced to the drug. Mass spectrometric detection of these modifications has been shown for tobramycin, kanamycin, and neomycin; however, since this method needs an instrument with high resolution [44], it has never been introduced to clinical microbiology laboratories using MALDI-TOF MS of a general grade.

### 4.3. Identification of the Modified Antimicrobial Target

Modified drug targets, which block the antimicrobial action of drugs resulting in AMR, are another object to detect using the MALDI-TOF MS methodology (Figure 2). Gram-negative bacterial lipid A is a target of polymyxins, and representative colistin resistance is conferred by lipid A modification. However, the only accredited and reproducible procedure to determine susceptibility to colistin is the broth microdilution method, and this technique takes days to obtain results and is extremely laborious [45]. As an alternative method, identification of colistin resistance in Gram-negative bacteria through the detection of modified lipid A using MALDI-TOF MS was tried [46]. Application of this method to *E. coli* [46] and to *Acinetobacter* spp. [47] presented promising results of a shift peak with *m*/*z* +123 Da for bis-phosphorylated hepta-acyl lipid A with reasonable sensitivity and specificity. While this method allowed the clear detection of colistin-resistant bacteria, a few limitations impede its wide use. For instance, the extra step to extract lipid A was complex [48], and for proper performance, the lipid A analysis needs to be conducted in negative ion mode in the range of 1000 *m*/*z* to 3000 *m*/*z*, requiring an instrument of a higher grade than the MALDI Biotyper of a general grade, such as MALDI-TOF/TOF MS [46]. Notably, the MALDIxin test, which enables the detection of colistin resistance by identifying the l-Ara4N- and pEtN-modified lipid A in Gram-negative bacteria using the MALDI Biotyper Sirius MALDI-TOF MS system, was developed [49].

rRNA methylation is one of the main causes of resistance to antimicrobial drugs that target the bacterial ribosome, such as macrolides, streptogramins, chloramphenicol, tetracyclines, and aminoglycosides. Various trials have been carried out to detect rRNA methylation using MALDI-TOF MS by detecting the +14 Da mass shift from the native ribosome peak of 999 *m*/*z* to a 1013 *m*/*z* peak from monomethylation or a 1027 *m*/*z* peak from dimethylation [50]. However, this method needs a difficult step to purify the bacterial ribosomes, and epoch improvement of the manual is needed for its popular use. Even though a simplified method was developed through the lipophylization of methylated ribosomes, it is still not practical for use as a rapid diagnostic method [51].

### 4.4. Direct Detection of the AMR Determinant

Direct detection of the proteins conferring AMR in bacterial hosts looks ideal, and the approach has been tried continuously (Figure 2). However, the trials were not always successful and even the developed method is not widely used in clinical microbiology laboratories due to several difficulties: the large molecular weights of the AMR determinants are not suitable for detection in the routine range of spectral determination used for species identification, and the low production of the AMR determinant proteins resulting in low intensities of the peaks issues indistinguishable peaks which almost contact to the baseline. Sophisticated protein analysis often requires the top-down strategy.

**Figure 3:**
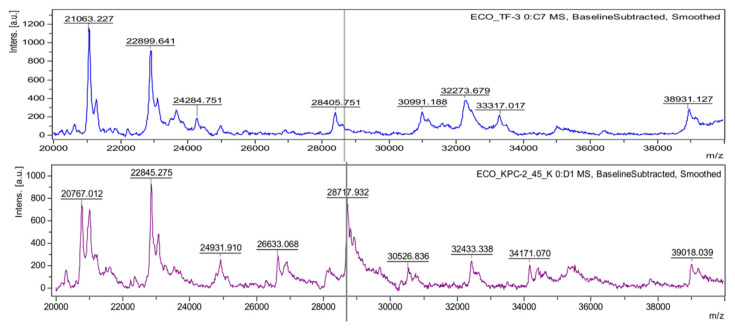
Identification of the KPC producer by using the KPC peak at 28718 *m*/*z*. Mass spectra were obtained from the lysates of drug-susceptible (**upper**) and carbapenem-resistant (**bottom**) *E. coli* conferred by the KPC-2 enzyme. A KPC peak is presented near the theoretical molecular weight of the KPC-2 polypeptide, with the gray vertical line, at 28718.13 *m*/*z*.

The first trial of the direct detection was for beta-lactamases produced by transformed *E.* *coli* grown in media with ampicillin [52]. The ideal straightforward approach was not simple to apply because the bacterial host does not express a large enough amount of the beta-lactamase gene, which is costly for the bacterial host. Thus, such AMR determinants often need preinduction to have enough of the protein to detect [53]. Only a few beta-lactamases, which are produced continuously without induction, presented successful results for the direct detection of AMR determinants using MALDI-TOF MS. A dominant penicillinase, TEM-1, which confers resistance to ampicillin in *E. coli*, was identified at 28972 *m*/*z* [52]. Additionally, a plasmid-mediated AmpC beta-lactamase (CMY-2) produced by clinical *E. coli* strains was detected by the MALDI-TOF MS method [54]. The molecular weight of the protein was 39.8 kDa after cleavage of the signal peptide, and the mass peak was identified at 39800 *m*/*z*. From the same approach, *K. pneumoniae* carbapenemase-2 (KPC-2) was detected at 28544 *m*/*z* [54]. A recent publication on the direct detection of the KPC protein suggested another mass peak at 28718 *m*/*z* (Figure 3), but this peak was likely due to the difference between the dominant subtypes of KPC at a specific region [55].

Porin defects, which are commonly associated with AMR or reduced antimicrobial susceptibility in Gram-negative bacteria, have been other targets to detect by MALDI-TOF MS [56]. Loss of OmpK36 in *K. pneumoniae* was detected by the method through disappearance of the peaks at 38000 *m*/*z* and 19000 *m*/*z* [57]. Overproduction of the efflux system is associated with multidrug resistance in Gram-negative bacteria and is a good subject to detect by MALDI-TOF MS. However, since this technique is not appropriate to quantify the protein accurately, the approach was not fruitful with MALDI-TOF MS but was possible with liquid chromatography (LC)-MS, such as LC coupled to an electrospray triple quadrupole MS [58].

### 4.5. Biomarkers That Are Coexpressed Proteins with AMR Determinants

As described in the above section, most of the proteins conferring AMR in the bacterial host have large molecular weights that are above the mass range of routine MALDI-TOF MS for species identification, which is between 2–20 kDa: the penicillin-binding protein 2a in *S. aureus*, approximately 76.4 kDa; beta-lactamases that are produced by Enterobacterales, such as TEM, SHV, CTX-M, and AmpC, approximately 30 kDa; 16S ribosomal methyltransferases conferring resistance to aminoglycosides in Gram-negative bacteria, such as ArmA and RmtA, approximately 30 kDa; and 23S ribosomal methyltransferases conferring macrolide-lincosamide-streptogramin B antimicrobials in Gram-positive bacteria, such as ErmA, ErmB, and ErmC, approximately 29 kDa [59]. Due to the narrow range available through the MALDI Biotyper and low resolution out of the usual range, alternative targets beyond the AMR determinants have been targeted and are often the protein that is coproduced in the antimicrobial-resistant bacteria (Figure 2).

Comparing MRSA and MSSA isolates, several peaks indicative of MRSA were able to be identified in the mass range of 500–3000 Da, but these peaks have never been further identified or characterized [60]. In 2014, a single peak at 2415 *m*/*z* was identified from a phenol extraction of MRSA isolates and was proposed to be an MRSA-specific phenol-soluble modulin (PSM)-*mec* peptide [61]. However, the 2415-*m*/*z* peak was identified in only the MRSA isolates carrying the SCC*mec* types II and III but not in the isolates harboring the SCC*mec* types I, IV, or XI, which is a great limitation as a biomarker to detect MRSA [62,63]. Similarly, a KPC-carrying plasmid-associated biomarker at 11109 *m*/*z* has been proposed for the identification of KPC-producing Enterobacterales [64]. However, *bla*_KPC_-carrying plasmid-associated biomarkers have very limited use in the detection of KPC-producing bacteria, since the *bla*_KPC_ gene is observed in divergent plasmids and sometimes in chromosomes [65,66,67].

## 5. Future Aspects

Through recent advances, MALDI-TOF MS-based approaches have exhibited considerable potential for the rapid, simple, and cost-effective diagnosis of AMR in microorganisms of clinical importance (Table 1). Present limitations of detection, especially when using the most common entry-level instrument for MALDI-TOF MS, include low resolution above and below the routine range of spectral determination used for species identification, and low-level expression of the AMR-determining proteins. However, varied approaches have been attempted to overcome these limitations and simple sample-processing methods and novel matrices are being developed to defeat the defects. Few exceptional discrepancies between the MALDI-TOF MS-based AMR determination and methods based on the AMR phenotypes of bacteria have been found, such as AMR conferred by overproduction of certain efflux systems [68] or by complete loss of the drug target [69], and applications of the MALDI-TOF MS method to a large set of clinical samples will help improvements in the method.

Beyond the rapid AMR detection, the MALDI-TOF MS methodology is able to have wider applications owing to its simple operation and availability for high-throughput systems. For instance, this methodology has been shown to be useful for the detection of protein toxins, such as staphylococcal enterotoxin B, botulinum neurotoxins, *Clostridium perfringens* epsilon toxin, and Shiga toxin, which can be potential bioterrorism agents when they are delivered via an aerosol route [77,78]. In addition, the identification of multiple bacteria in complex polymicrobial mixtures [79] is another aspect we expect to be developed through this methodology.

Some manipulations are needed to enhance the applications of MALDI-TOF MS for AMR diagnostics. Primarily, the instrument quality is diverse, those with high specifications are very expensive, and unfortunately, the spectra produced by instruments with different specifications are often incompatible. Concentrated research and development based on continuous trials is needed to overcome the issue, especially to use the top-down approach to identify specific markers indicating the AMR [55,80].

## 6. Conclusions

Thanks to the development of the MALDI-TOF MS technique, the identification of organisms causing infectious diseases is no longer a time-determining step for optimizing a treatment strategy. In addition to microorganism species identification, this methodology can also be the leading application for the identification of AMR in bacterial pathogens in clinical settings. In particular, its great speed and simple application would be the most suitable for endemic AMR clinical strains in specific settings, i.e., MRSA, VRE, carbapenem-resistant *A. baumannii* and *P. aeruginosa*, and ESBL-, AmpC-, and carbapenemase-producing Enterobacterales. More effort should be made to develop detection methods for drug-resistant bacterial pathogens.

## Figures and Tables

**Table 1:** MALDI-TOF MS-based rapid diagnostic methods for AMR.

Strategy	Target	Target Resistance	Commercialized (Company) and/or Developing Products	Ref.
Antimicrobial susceptibility testing	AUC ^1^ shift spectra (isotope)	All antimicrobials	MBT-ASTRA (Bruker) MBT-Resist (Bruker) DoT-MGA ^2^ (inv. ^3^)	[22,24,25,70,71]
ID of the AMR clonal group	Clone-specific spectra	Methicillin (MRSA)	ClinProtTools (Bruker)	[72]
ID of the modified antimicrobial drug	Shift peak of the modified drug	Beta-lactams Cephalosporins Carbapenems	MBT STAR^®^-BL (Bruker) MBT STAR^®^-Cepha (Bruker) MBT STAR^®^-Carba (Bruker)	[40,73,74]
ID of the modified antimicrobial target	Shift peak of the modified drug target	Colistin	MALDIxin test (inv.)	[49,75]
Direct detection of the AMR determinant	Protein-specific peak	Carbapenems	Aprodete^TM^ KPC-CPE (Aprilis)	[55]
Biomarker detection	Protein-specific peak	Carbapenems	ClinProtTools (Bruker)	[64,76]

^1^ AUC, area under curve. ^2^ DoT-MGA, Direct-on-Target Microdroplet Growth Assay. ^3^ inv., under investigation.

## Data Availability

All data generated or analyzed during this study have been included in this article.
